# Complete response of squamous cell carcinoma of the lung following treatment with pembrolizumab in an elderly patient: A case report

**DOI:** 10.1111/1759-7714.13733

**Published:** 2020-11-10

**Authors:** Eiji Takeuchi, Yuri Okamoto, Naoki Takahashi, Shun Morizumi, Yuko Toyoda, Naoto Kuroda, Kenji Yorita

**Affiliations:** ^1^ Department of Clinical Investigation National Hospital Organization Kochi Hospital Kochi Japan; ^2^ Department of Respiratory Medicine Japanese Red Cross Kochi Hospital Kochi Japan; ^3^ Department of Respiratory Medicine and Rheumatology Tokushima University Graduate School of Biomedical Sciences Tokushima Japan; ^4^ Department of Internal Medicine Japan Agricultural Cooperatives Kochi Hospital Nankoku Japan; ^5^ Medical Office Kobe Kyodo Hospital Kobe Japan; ^6^ Department of Diagnostic Pathology Japanese Red Cross Kochi Hospital Kochi Japan

**Keywords:** Complete response, elderly patient, first‐line, non–small cell lung cancer, pembrolizumab

## Abstract

Complete response of non–small cell lung cancer (NSCLC) with immune checkpoint inhibitor (ICI) monotherapy is rare. Here, we encountered an elderly patient who showed complete response of NSCLC following treatment with pembrolizumab. An 84‐year‐old man with a history of bloody sputum for several weeks visited a general physician. At that time, a chest X‐ray revealed a tumor shadow in the left middle lung field, and the patient was referred to our hospital. Following transbronchial biopsy, he was diagnosed with squamous cell carcinoma of the lung. Expression of programmed death ligand 1 (PD‐L1) in tumor cells was 80% or more by immunostaining. Based on the above, immunotherapy with pembrolizumab was performed as first‐line therapy. The cancer cells completely disappeared at the end of the fifth cycle. There were no side effects during the therapeutic course. Treatment with pembrolizumab continued for two years and was then discontinued at the patient's request. Since then, no tumor recurrence has been detected for about one and a half years without treatment. There have been few reports of lung cancer disappearing after treatment with pembrolizumab. In conclusion, in elderly NSCLC patients with PD‐L1 expression of 50% or more, pembrolizumab should be considered as first‐line treatment with the treatment period, and mechanism suggested in this report.

## Graphical abstract

Here, we present a case of an elderly patient who had a complete response of non–small cell lung cancer (NSCLC) following treatment with pembrolizumab. The complete response was maintained for one and a half years after discontinuation of two years of treatment. In elderly patients with NSCLC with PD‐L1 expression of more than 50%, pembrolizumab should be considered as first‐line treatment with the treatment period, and mechanism suggested in this report.

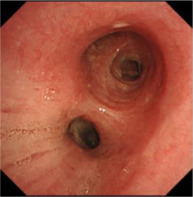

## Introduction

Lung cancer is the leading cause of cancer death worldwide. However, over the past decade, the treatment paradigm, especially immunotherapy against lung cancer, has markedly evolved. In patients with advanced non–small cell lung cancer (NSCLC) and programmed death ligand 1 (PD‐L1) expression of at least 50% of tumor cells, pembrolizumab has been reported to achieve significantly longer progression‐free and overall survival and fewer adverse events than platinum‐based chemotherapy (KEYNOTE‐024).^1^

Here, we present and describe the case of an elderly patient who showed a complete response (CR) of NSCLC following treatment with pembrolizumab.

## Case report

**Figure 1:**
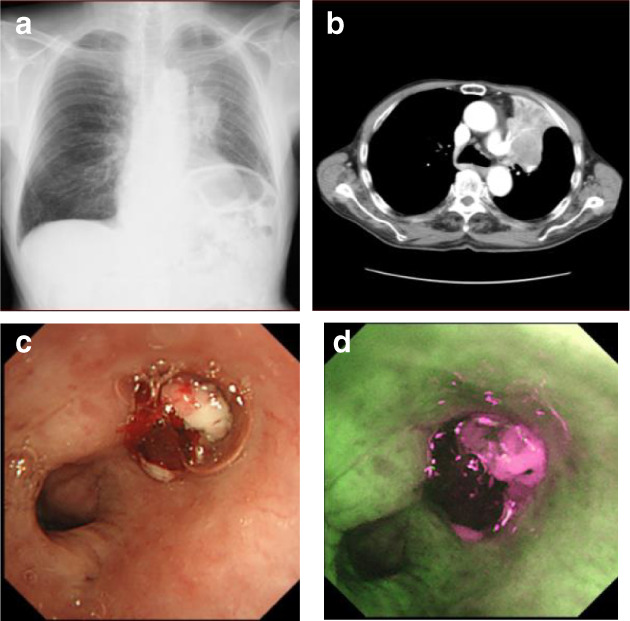
(**a**) Chest X‐ray and (**b**) contrast‐enhanced computed tomography (CT) at first admission. Bronchoscopy with (**c**) standard and (**d**) fluorescent observations revealed a tumor obstructing the left upper lobe.

**Figure 2:**
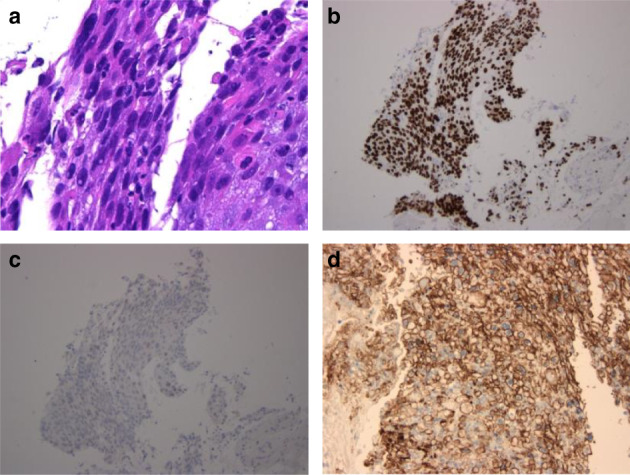
(**a**) Hematoxylin and eosin‐stained section showed a sheet of atypical spindle cells (magnification ×400). (**b**–**d**) By immunostaining, the tumor cells were diffusely positive for p40 (magnification ×100,**b**), negative for TTF1 (magnification ×100,**c**), and positive for programmed death ligand 1 (≥80%, magnification ×200,**d**).

**Figure 3:**
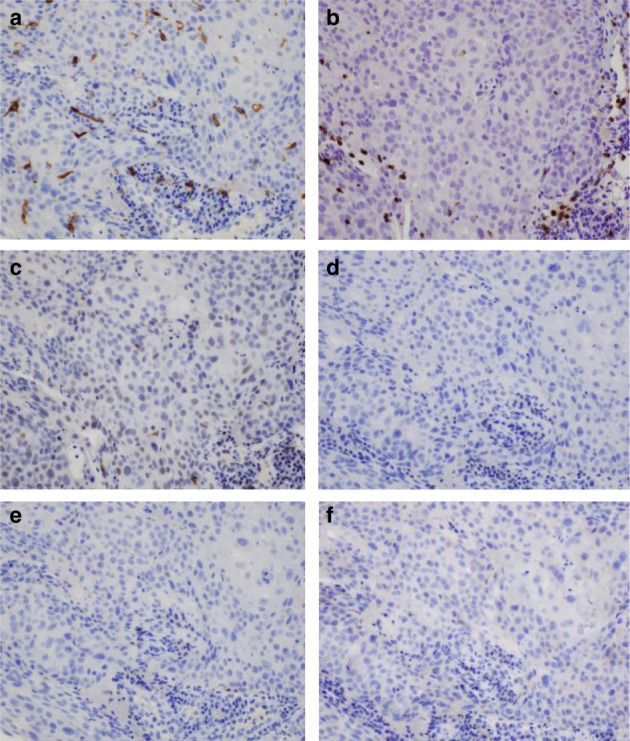
Immunostaining of the tumor cells. (**a**) A carcinoma with high density of CD163‐positive TAM in tumor stroma (magnification ×400). (**b**) A carcinoma with high density of MPO‐positive TAN in and around the tumor (magnification ×400). (**c**) A carcinoma with high density of CD25‐positive Treg in the tumor (magnification ×400). (**d**–**f**) A carcinoma with low density of CD4‐positive T cell (magnification ×400, **d**), CD8‐positive T cell (magnification ×400, **e**), and CD56‐positive NK cell (magnification ×400, **f**).

An 84‐year‐old man complained of bloody sputum for several weeks and visited a general physician. At that time, a chest X‐ray revealed a tumor shadow in the left middle lung field, and the patient was referred to our hospital. He had been smoking two packets of cigarettes per day for 20 years. His Eastern Cooperative Oncology Group performance status (ECOG PS) was 1. Blood tests showed a high serum level of cytokeratin 19 fragment (5.7 ng/mL). Neutrophil‐to‐lymphocyte ratio was 2.35. Chest X‐ray (Fig 1a) and contrast‐enhanced computed tomography (CT) (Fig 1b) showed a tumor in the left upper lobe, which invaded the pulmonary artery. Bronchoscopy revealed that a tumor was obstructing the upper left lobe (Fig 1c,d). Transbronchial biopsies showed atypical polygonal spindle cells arranged in a sheet (Fig 2a). A bridge between cells was observed. Tumor cells were diffusely positive for p40 (Fig 2b) by immunostaining. Similarly, tumor cells were negative for TTF‐1 (Fig 2c). Expression of PD‐L1 in tumor cells accounted for more than 80% by immunostaining (Fig 2d). Further immunostaining revealed many CD163‐positive tumor‐associated macrophages (TAMs) (Fig 3a), CD15, myeloperoxidase (MPO)‐positive tumor‐associated neutrophils (TANs) (Fig 3b), and CD3, CD25‐positive regulatory T cells (Tregs) in the tumor (Fig 3c). The number of CD4 (Fig 3d), CD8 (Fig 3e), and CD56 (Fig 3f) positive cells was minimal. No other distant metastases were seen, and the patient was clinically diagnosed with left upper lobe squamous cell carcinoma cT4N2M0 stage IIIB.

**Figure 4:**
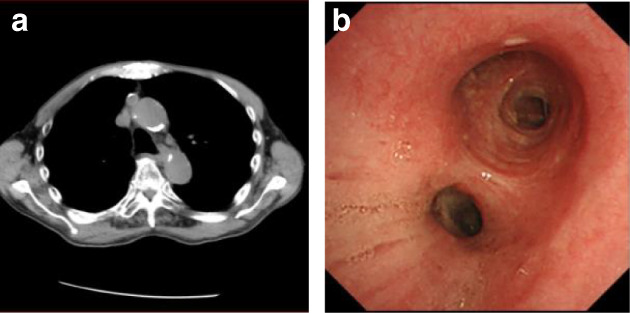
(**a**) Computed tomography (CT) and (**b**) bronchoscopy after pembrolizumab treatment. The tumor had completely disappeared at the end of the fifth cycle.

Radiation therapy may lead to sudden death due to perforation of the pulmonary artery caused by pulmonary artery infiltration of the tumor,^2^ and the patient chose not to undergo this treatment approach. Therefore, immunotherapy with pembrolizumab was commenced at three‐week intervals because PD‐L1 expression in the tumor cells was 80% or more. The cancer cells had completely disappeared at the end of the fifth cycle (Fig 4a,b). There were no side effects during the therapeutic course. Tumor markers were also normalized and maintained. After the treatment continued for two years, the procedure was discontinued at the request of the patient. Since then, the tumor has not recurred for about one and a half years without treatment.

## Discussion

In this report, we encountered an elderly patient showing CR of NSCLC following treatment with pembrolizumab. The CR was maintained for one and a half years following discontinuation of two years of treatment. To the best of our knowledge, CR of NSCLC after treatment with pembrolizumab is extremely rare. We were only able to find two previous case reports.^3^, ^4^ Similarly, a few cases show the effectiveness of nivolumab against lung cancer; and we found three such case reports.^5^, ^6^, ^7^ In the CheckMate 057 trial, nivolumab achieved four points of CR out of 292 cases.^8^ In the OAK study, atezolizumab achieved CR in six out of 425 patients.^9^ In the KEYNOTE‐024 trial, CR was found in only six out of 154 patients (3.8%). A meta‐analysis of clinical trials also reported that the ratio was low (only 1.5%), but immune checkpoint inhibitors (ICIs) significantly increased this ratio compared with chemotherapy.^10^

Immunostaining revealed many CD163‐positive TAMs (M2 macrophages), CD15, MPO‐positive TANs, and CD3, CD25‐positive Tregs in the tumor. Surprisingly, the number of CD4, CD8, and CD56 positive cells was minimal. M2 macrophages, Treg, and TAN each express PD‐L1 and induce immunosuppression at the tumor site. ^11^, ^12^, ^13^ Pembrolizumab may overcome these tumor immunosuppressive states.

Age‐related changes in the immune system of elderly patients may lead to a decline in immune function; therefore, such patients may benefit little from ICIs. The U.S. Food and Drug Administration analyzed survival in older adults with metastatic NSCLC in controlled trials of ICIs.^14^ Patients aged 65 and older with advanced and metastatic NSCLC, including those ≥75 years of age, seem to have similar survival benefits from treatment with ICIs as patients <65 years of age.

Although it is reportedly better to maintain ICIs, all patients who achieved CR were long‐term survivors and had no signs of disease relapse.^15^ However, there is no clear evidence on how long treatment should continue.^16^, ^17^ Other studies have reported that the drug was effective once it was discontinued and readministered when it recurred.^18^, ^19^ If the tumor completely disappears as in the present case, then it may be possible to administer pembrolizumab for a certain period, discontinue it, and if cancer recurs, re‐challenge it. Clinical trials are currently underway in Japan.^20^

In conclusion, in this study, we present a case of an elderly patient showing CR of NSCLC following treatment with pembrolizumab. The CR was maintained for one and a half years after discontinuation of two years of treatment. There have been few reports of lung cancer disappearing after treatment with pembrolizumab, and the clinical course in the present case was uneventful without any marked side effects. In elderly patients with NSCLC with PD‐L1 expression of more than 50%, pembrolizumab should therefore be considered as first‐line treatment with the treatment period, and mechanism suggested in this report.

## Disclosure

The authors declare that there are no conflicts of interest.
